# Discovery of genetic susceptibility variants in pediatric and adult ependymoma

**DOI:** 10.1093/noajnl/vdag004

**Published:** 2026-01-16

**Authors:** Joshua D Strauss, Priya B Shetty, Spiridon Tsavachidis, Jinyoung Byun, Stephen C Mack, Xiao Xiangjun, Terri S Armstrong, Mark R Gilbert, (on behalf of NCI-CONNECT), Lisa Mirabello, Smita Bhatia, Wendy M Leisenring, Lindsay M Morton, Gregory T Armstrong, Jon Foss-Skiftesvik, Christian Munch Hagen, Jonas Bybjerg-Grauholm, iPSYCH Consortium, Manel Ghozal, Audrey Bonaventure, Jacqueline Clavel, Melissa L Bondy, (on behalf of GICC), Christopher I Amos, Thanh T Hoang, Michael E Scheurer

**Affiliations:** Center for Epidemiology and Population Health, Department of Pediatrics, Baylor College of Medicine, Houston, Texas; Department of Pediatrics, Division of Hematology-Oncology, Baylor College of Medicine, Houston, Texas; Texas Children’s Cancer Center, Texas Children’s Hospital, Houston, Texas; Department of Medicine, Section of Epidemiology and Population Sciences, Baylor College of Medicine, Houston, Texas; Dan L. Duncan Comprehensive Cancer Center, Baylor College of Medicine, Houston, Texas; Department of Medicine, Section of Epidemiology and Population Sciences, Baylor College of Medicine, Houston, Texas; Division of Epidemiology, Biostatistics, and Preventive Medicine, Department of Internal Medicine, University of New Mexico School of Medicine, Albuquerque, New Mexico; Developmental Neurobiology, St Jude Children’s Research Hospital, Memphis, Tennessee; O’Neal Comprehensive Cancer Center, University of Alabama at Birmingham, Birmingham, ­Alabama; Department of Medicine, Section of Epidemiology and Population Sciences, Baylor College of Medicine, Houston, Texas; Dan L. Duncan Comprehensive Cancer Center, Baylor College of Medicine, Houston, Texas; Institute for Clinical and Translational Research, Baylor College of Medicine, Houston, Texas; Center for Cancer Research, National Cancer Institute, Bethesda, Maryland; Center for Cancer Research, National Cancer Institute, Bethesda, Maryland; Division of Cancer Epidemiology and Genetics, National Cancer Institute, National Institutes of Health, Rockville, Maryland; Clinical Research Division, Fred Hutchinson Cancer Research Center, Seattle, Washington; Division of Cancer Epidemiology and Genetics, National Cancer Institute, National Institutes of Health, Rockville, Maryland; Epidemiology and Cancer Control, St Jude Children’s Research Hospital, Memphis, Tennessee; Department of Neurosurgery, Rigshospitalet University Hospital, Copenhagen, Denmark; Danish Center for Neonatal Screening, Department of Congenital Diseases and Neonatal Genetics, Statens Serum Institut, Copenhagen, Denmark; Department of Neurosurgery, Rigshospitalet University Hospital, Copenhagen, Denmark; Danish Center for Neonatal Screening, Department of Congenital Diseases and Neonatal Genetics, Statens Serum Institut, Copenhagen, Denmark; The Lundbeck Foundation Initiative for Integrative Psychiatric Research (iPSYCH); Center for Research in Epidemiology and Statistics CRESS, Epidemiology of Childhood and Adolescent Cancer Team, University Paris Cité, University Paris Sorbonne Nord, INSERM, INRAe, Villejuif, France; The Lundbeck Foundation Initiative for Integrative Psychiatric Research (iPSYCH); Center for Research in Epidemiology and Statistics CRESS, Epidemiology of Childhood and Adolescent Cancer Team, University Paris Cité, University Paris Sorbonne Nord, INSERM, INRAe, Villejuif, France; The Lundbeck Foundation Initiative for Integrative Psychiatric Research (iPSYCH); Center for Research in Epidemiology and Statistics CRESS, Epidemiology of Childhood and Adolescent Cancer Team, University Paris Cité, University Paris Sorbonne Nord, INSERM, INRAe, Villejuif, France; National Registry of Childhood Cancer, Hôpital Paul Brousse, Groupe Hospitalier Universitaire Paris-Sud, Assistance Publique Hôpitaux de Paris (AP-HP), Villejuif, Vandoeuvre-lès-Nancy, France; Centre Hospitalier Régional Universitaire de Nancy, Vandoeuvre-lès-Nancy, France; Department of Epidemiology and Population Health, Stanford University School of Medicine, Stanford, California; Division of Epidemiology, Biostatistics, and Preventive Medicine, Department of Internal Medicine, University of New Mexico School of Medicine, Albuquerque, New Mexico; Center for Epidemiology and Population Health, Department of Pediatrics, Baylor College of Medicine, Houston, Texas; Department of Pediatrics, Division of Hematology-Oncology, Baylor College of Medicine, Houston, Texas; Texas Children’s Cancer Center, Texas Children’s Hospital, Houston, Texas; Dan L. Duncan Comprehensive Cancer Center, Baylor College of Medicine, Houston, Texas; Department of Pediatrics, Emory University School of Medicine, Atlanta, Georgia

**Keywords:** central nervous system tumor, ependymoma, genome-wide association study, germline, single nucleotide polymorphism

## Abstract

**Background:**

Ependymoma is a malignancy of the neuroepithelium-derived ependyma that lines the spinal cord and ventricles of the brain, occurring most frequently in young children and older adults. Genetic susceptibility to ependymoma has proven difficult to assess due to disease rarity.

**Methods:**

We performed genome-wide association studies (GWAS) of 478 ependymoma patients and 4,841 disease-free controls of European ancestry. Ependymoma patients consisted of 117 children (<18 years old) with whole-genome sequencing (WGS), 142 children with genotyping, and 219 adults (≥18 years old) with genotyping. Genotyped samples were imputed using the 1,000 Genomes Project as the reference panel and underwent quality control filtering. The GWAS was performed separately by age group and technology (genotyped or WGS). GWAS variants were considered significant at *P* < 5 × 10^−8^.

**Results:**

Among pediatric subjects with WGS data, we identified a significant intronic variant in *EDIL3* (rs149378, *P* = 1.9 × 10^−8^) and a nearly significant intronic variant in *LHX4* (rs79008224, *P* = 7.2 × 10^−8^). In pediatric subjects with genotyped data, two significant intronic variants were detected: *FAM149A* (rs6852180, *P* = 1.8 × 10^−8^) and *CYS1* (rs61052588, *P* = 3.0 × 10^−8^). Additionally, an intergenic variant near *C1orf94* (rs1404350, *P* = 1.2 × 10^−14^) was highly significant. In genotyped adult subjects, a single variant was observed in *KCNQ3* (rs79089725, *P* = 2.0 × 10^−8^).

**Conclusion:**

Our analysis represents one of the most extensive ependymoma-specific GWAS conducted to date. Several significant intronic variants were harbored in genes associated with cancer and neurological disease. Future studies are needed to investigate the role of these age-specific alterations in ependymoma pathogenesis.

Key Points

- This study provides an extensive ependymoma susceptibility analysis
- *CYS1*, *EDIL3*, and *FAM149A* were involved in pediatric ependymoma susceptibility
- *KCNQ3* harbored a variant associated with adult ependymoma

Importance of the Study

Ependymoma is a malignancy of the ependyma that lines the spinal cord and ventricular system of the brain. Due to its rarity, the contribution of germline variants to ependymoma risk has been difficult to assess. Our genome-wide association studies (GWAS) included 259 pediatric (<18 years old) and 219 adult (≥18 years old) ependymoma patients using 4,841 disease-free controls with genotyping or whole genome sequencing data. In the pediatric cases, significant intronic variants were identified in *CYS1* (rs61052588, *P* = 3.0 × 10^−8^), *EDIL3* (rs149378, *P* = 1.9 × 10^−8^), *FAM149A* (rs6852180, *P* = 1.8 × 10^−8^), and *LHX4* (rs79008224, *P* = 7.2 × 10^−8^), as well as an intergenic variant near *C1orf94* (rs1404350, *P* = 1.2 × 10^−14^). In adults, one significant variant was found in *KCNQ3* (rs79089725, *P* = 2.0 × 10^−8^). Many of the genes harboring significant GWAS variants have been implicated in neurologic disorders or cancer. This analysis represents one of the most extensive ependymoma-specific GWAS to date and may serve as a basis for future analysis into genetic susceptibility to ependymoma.

Ependymoma is a rare malignancy of the central nervous system (CNS) arising from the neuroepithelium-derived ependyma that lines the spinal cord and ventricles of the brain, with an annual incidence of approximately two cases per million individuals in the United States. Under normal conditions, the glial cell-derived ependyma regulates cerebrospinal fluid and supports neurogenesis.^1^^,^^2^ Ependymomas can occur at any age, but incidence primarily follows a biphasic distribution with peaks in children under 10 years old and adults in their 50s.^3^^,^^4^ Disease location typically differs by age, with ∼90% of pediatric cases occurring in the brain, whereas two-thirds of adult cases occur in the spinal cord.^5^

Germline variation related to ependymoma has not been thoroughly investigated. Previous studies have identified ependymoma susceptibility alterations in *APC*, *NF1*, *NF2*, *LTZR1*, *PMS2*, and *TP53*.^6–8^ However, these investigations were limited by the number of ependymoma patients included, absence of disease-free controls, and were limited to known cancer-related genes. Genome-wide association studies (GWAS) of ependymoma may identify novel susceptibility loci that provide insight into tumor development. Considering the approximately 40-year difference between the most common ages of disease occurrence, we posit that there are several shared and distinct germline variants by age of diagnosis. The genetic assessment of ependymoma susceptibility is critical to advance our comprehension of disease etiology and early detection techniques.

In this study, we performed GWAS of adult and pediatric ependymoma patients to identify novel disease-associated common germline variants.

## Methods

### Data Sources

Ependymoma patients and controls with genotyping or whole genome sequencing (WGS) data were identified from several consortia/studies. A description of the studies and technologies used is available in the Supplementary Material (S1).

### Ethics

Each original study collected participants’ clinical information and samples for DNA extraction after obtaining written informed consent on protocols individually approved by each study’s ethics review board. The current GWAS analysis was approved by the Baylor College of Medicine (Houston, TX) Institutional Review Board (IRB).

### Whole-Genome Sequencing Calling

WGS samples aligned to Genome Reference Consortium Human Build 38 (GRCh38) were processed according to the GATK v4.5.0 germline short variant discovery best practices workflow.^9^ All sample files were jointly called to enhance the sensitivity and accuracy of variant detection. Called samples were then hard filtered with the following GATK suggested thresholds: quality score > 30, quality normalized by depth > 2, mapping quality > 40, mapping quality rank sum > −12.5, read position rank sum > −8, symmetric odds ratio < 3.0, Fisher strand bias < 60. Samples with a call rate < 95% were removed.

### Genotyped Sample Imputation

Genotyped samples were imputed to improve variant overlap between multiple study methodologies and genomic platforms. First, samples with a call rate < 95%, structural variants, and multi-allelic variants were removed from further analyses. Samples were all lifted over to GRCh38 using CrossMap v0.7.0 and aligned to the 30X high-coverage 1000 Genomes Project (1KG) GRCh38 reference panel of 2,504 unrelated samples.^10^^,^^11^ Phasing and imputation were performed using BEAGLE 5.4 with 1KG as the reference panel.^12^^,^^13^ Imputed single-nucleotide polymorphisms (SNP) were filtered by imputation quality score (dosage-*R*^2^ > 0.4).

### Quality Control

Samples were merged into three analytic sets based on age group (pediatric or adult) and technology (genotyping or WGS). First-degree related sample pairs were identified using Kinship-based INference for Gwas (KING) in Plink2, with one sample of each pair being randomly removed from further analyses, if present.^14^^,^^15^ SNPs with call rates <98%, minor allele frequency (MAF) < 5%, or a significant departure from Hardy-Weinberg equilibrium (*P* < 1 × 10^−20^) in controls were excluded. The ancestry of all samples was genomically inferred into one of five distinct super-populations (Admixed American, African, East Asian, European, South Asian) using KING v2.3.2 with the known ancestries from 1KG samples. Data transformation and quality control measures were performed with BCFtools and Plink2.^16^

### Study Population

Ependymoma patients met diagnostic coding requirements according to the International Classification of Childhood Cancer, third edition (ICCC-3; code IIIa) or the International Classification of Diseases for Oncology, third edition (ICD-O-3; histology codes 9383 and 9391-9394) for pediatric and adult patients, respectively.^17^^,^^18^ A total of 478 patients diagnosed with ependymoma and 4,841 disease-free controls of genomically inferred European ancestry with germline genotyped or sequencing data passed the quality control methods described above. Of the 478 patients, 259 were children (<18 years old) and 219 were adults (≥18 years old). Disease-free controls had no record of CNS malignancies in their respective studies. Cases and controls were assigned into three sets, consisting of pediatric subjects with WGS (117 cases, 285 controls), pediatric subjects with genotyping (142 cases, 1331 controls), and adult subjects with genotyping (219 cases, 3225 controls; Table 1). The multi-ancestral GWAS of the pediatric subjects with genotyping and pediatric subjects with WGS is available in the Supplementary Materials (S2-S4) and Supplementary Tables.

**Table 1.** Summary of genome-wide association study cases and controls with European ancestry

Age group	Genomic analysis	Case/control	Cohort/study	Subjects pre-QC	Subjects post-QC	Sex-male post-QC
**Pediatric**	Whole genome sequencing	Case	CBTN	48	48	56%
			St. Jude	73	69	
		Control	St. Jude	298	285	48%
	Genotyping	Case	ACCESS	60	60	64%
			CCSS	55	55	
			GICC	8	8	
			NCI-Connect	9	8	
			TOPNOC	12	11	
		Control	ADD Health	1340	1331	47%
**Adult**	Genotyping	Case	ACCESS	6	6	40%
			GICC	77	73	
			NCI-Connect	157	140	
		Control	GICC	3249	3225	57%

Abbreviations: ACCESS, adolescent and childhood cancer epidemiology and susceptibility service of Texas; ADD Health, national longitudinal study of adolescent health; CBTN, childhood brain tumor network; CCSS, childhood cancer survivor study; GICC, glioma international case control consortium; NCI-Connect, national cancer institute’s program for the comprehensive oncology network evaluating rare CNS tumors; St Jude, St Jude cloud; TOPNOC, Texas-Oklahoma pediatric neuro-oncology consortium; QC, quality control.

### Statistical Analysis

#### Genome-wide analyses

Ependymoma occurrence was modeled for each variant using a generalized linear mixed model association test (GMMAT) in R v4.4.1 (R Foundation for Statistical Computing). GMMAT v1.4.2 was implemented to adjust for population structure and cryptic relatedness.^19^ The genetic relationship matrix required for GMMAT was created using Plink2. WGS and genotyped models were adjusted for the first 2 principal components (PC) and 6 PCs, respectively. Genome-wide significant variants (*P* < 5 × 10^−8^) were assessed for independence by evaluating linkage disequilibrium between adjacent (±250 kilobases) SNPs (*r*^2^ < 0.6), and resulting significant and independent SNPs were annotated with ANNOVAR.^20^ Summary Manhattan and quantile-quantile (QQ) plots were created with GWASLab v3.5.7.^21^ GWAS model fit was assessed using the genomic inflation factor (*λ*_GC_) and QQ-plots.

## Results

### Pediatric Variant Analysis

**Figure 1.**
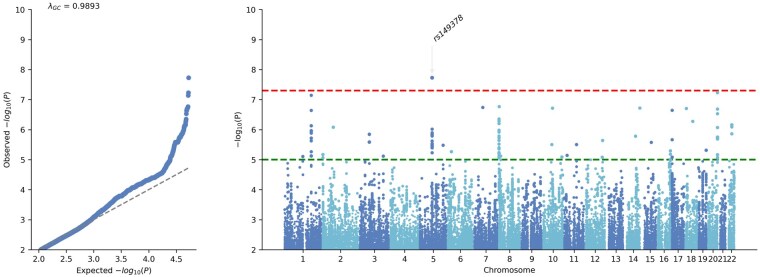
Genome-wide association study of whole-genome sequenced pediatric ependymoma of European ancestry. Each genome-wide association study displays the corresponding quantile-quantile plot (left) and Manhattan plot (right). The red upper dotted horizontal line indicates the genome-wide significance threshold (*P* = 5 × 10^−8^), and the green lower dotted line marks the suggestive significance threshold (*P* = 1 × 10^−5^).

The GWAS of pediatric subjects with WGS assessed 5.8 million variants. Of these, one variant was independent and significant (Figure 1, Table 2, Supplementary Tables). This intronic variant was present in *EDIL3* (rs149378, *P* = 1.9 × 10^−8^). A nearly significant supported intronic variant was harbored in *LHX4* (rs79008224, *P* = 7.2 × 10^−8^). Cytogenetic band 20q13.33 also displayed a notable signal with two independent variants in an intron of *COL9A3* (rs73157303, *P* = 3.0 × 10^−7^) and downstream (660 bases) of *OGFR* (rs910151, *P* = 5.9 × 10^−8^).

**Table 2.** Significant and independent single-nucleotide polymorphisms from genome-wide association studies

Analytic set	CHR	BP	rsID	A1	A2	Case genotypes	Control genotypes	Case AF	Control AF	Cases	Controls	Score	P-value	Band	Feature	Gene
Pediatric with genotyping	1	34299373	rs1404350	C	A	7-21-114	5-153-1173	0.123	0.061	142	1331	-11.614	1.23E-14	1p35.1	intergenic	C1orf94 (distance = 80243 BP)
Pediatric with genotyping	2	10070302	rs61052588	C	T	16-57-69	75-516-740	0.313	0.250	142	1331	-9.072	3.02E-08	2p25.1	intronic	CYS1
Pediatric with genotyping	4	186171576	rs6852180	G	A	1-18-123	5-133-1193	0.070	0.054	142	1331	-4.844	1.81E-08	4q35.1	intronic	FAM149A
Pediatric with WGS	5	84182729	rs149378	C	T	34-57-26	44-146-95	0.534	0.411	117	285	-19.705	1.88E-08	5q14.3	intronic	EDIL3
Adult with genotyping	8	132455457	rs79089725	T	A	3-27-189	8-362-2855	0.075	0.059	219	3225	-15.642	2.04E-08	8q24.22	intronic	KCNQ3

Abbreviations: A1, test allele; A2, reference allele; AF, allele frequency; BP, base pair; CHR, chromosome; WGS, whole genome sequencing.

Case/Control Genotypes—Number of individuals with genotype (A1/A1-A1/A2-A2/A2).

**Figure 2.**
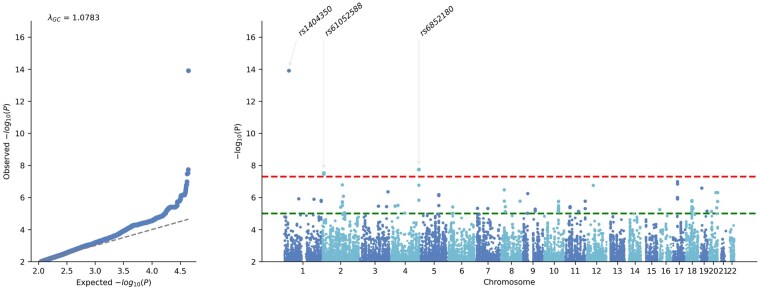
Genome-wide association study of genotyped pediatric ependymoma of European ancestry. Each genome-wide association study displays the corresponding quantile-quantile plot (left) and Manhattan plot (right). The red upper dotted horizontal line indicates the genome-wide significance threshold (*P* = 5 × 10^−8^), and the green lower dotted line marks the suggestive significance threshold (*P* = 1 × 10^−5^).

Pediatric subjects with genotyping GWAS assessed 4.8 million variants. A total of three SNPs were considered independent and significant (Figure 2, Table 2, Supplementary Tables). Intronic variants were found in *FAM149A* (rs6852180, *P* = 1.8 × 10^−8^) and *CYS1* (rs61052588, *P* = 3.0 × 10^−8^). Additionally, an intergenic SNP was adjacent (80 kilobases) to gene *C1orf94* (rs1404350, *P* = 1.2 × 10^−14^).

### Adult Variant Analysis

**Figure 3.**
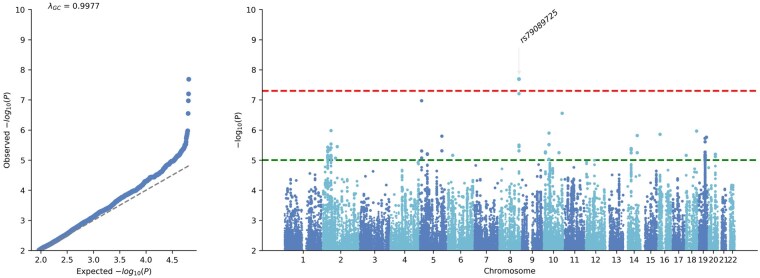
Genome-wide association study of genotyped adult ependymoma of European ancestry. Each genome-wide association study displays the corresponding quantile-quantile plot (left) and Manhattan plot (right). The upper red dotted horizontal line indicates the genome-wide significance threshold (*P* = 5 × 10^−8^), and the lower green dotted line marks the suggestive significance threshold (*P* = 1 × 10^−5^).

The adult subjects with genotyping set assessed 5.9 million variants. One SNP was significant and independent (Figure 3, Table 2, Supplementary Tables). This variant was harbored in the intron of *KCNQ3* (rs79089725, *P* = 2.0 × 10^−8^).

## Discussion

Ependymoma’s rarity impedes research into advancing knowledge of disease etiology and detection techniques. Our analysis represents the most extensive ependymoma-specific GWAS conducted to date and identifies several novel susceptibility loci unique to pediatric and adult cases.

### Pediatric Ependymoma Variants

Multiple pediatric-specific loci and disease-associated genes were linked to ependymoma risk. The GWAS conducted in pediatric individuals with WGS data identified a significant association with *EDIL3*. Several cancers have been associated with *EDIL3*, such as breast, gastric, liver, and lung cancer.^22–25^ Of relevance, EDIL3 variants have been reported as potentially pathogenic in desmoplastic infantile astrocytoma and ganglioglioma, and the gene may contribute to pathways involved in Alzheimer’s disease pathogenesis.^26^^,^^27^ Notable signals were also present in the introns of *LHX4* and *COL9A3*, as well as downstream of *OGFR*. *LHX4* is involved in neural development, primarily in the pituitary region, and has been linked with colorectal cancer.^28^^,^^29^ A recent ependymoma study using a murine model identified *LHX4* and *LHX2* enrichment.^30^  *COL9A3* has also been directly linked to ependymoma tumor invasiveness and structural development of the RELA molecular tumor subtype.^31^^,^^32^  *OGFR* has not previously been associated with ependymoma but is well known for the gene’s role in several malignancies as well as CNS function.^33–35^

In pediatric subjects analyzed by genotyping, significant variants were found in *CYS1* and *FAM149A*. *CYS1* has been associated with glioma tumorigenesis and survival.^36^^,^^37^  *FAM149A* has been identified as a potential prognostic marker in glioblastoma survival.^38^ A significant intergenic variant was located ∼80 kilobases from *C1orf94,* which, although largely uncharacterized, has been associated with epilepsy and glioblastoma.^39^^,^^40^

### Adult Ependymoma Variants

The adult GWAS identified a single significant intronic variant in *KCNQ3*, a gene that has been well established for its role in epilepsy and developmental disorders, such as autism.^41^^,^^42^  *KCNQ3* has been linked to metastasis in thyroid and esophageal cancer, and emerging evidence suggests it may also contribute to broader oncogenic processes and represents a potential therapeutic target.^43–45^

### Strengths and Limitations

Key strengths of this study include its unprecedented sample size, nearly 500 patients pooled from multiple consortia, and analytic separation by age group. Employing both sequencing and imputed genotyping analyses strengthened the robustness of the findings. Rigorous statistical methodologies further support the validity of the associations.

Limitations to note include heterogeneity introduced by multiple genetic platforms and study methodologies, which may increase susceptibility to bias or confounding. Importantly, we did not observe shared significant variants between pediatric and adult analytic sets, highlighting possible biologic differences. Restriction to individuals of ­European ancestry limits generalizability to other ancestries. As our analysis concentrated on common SNPs, the effect of structural variants and rare mutations remains unknown. Finally, molecular and anatomical tumor subtypes, which also vary by sex, were unavailable, precluding analysis of clinical heterogeneity.

In summary, nearly 500 patients from several consortia contributed to our findings, yielding the most extensive ependymoma-specific GWAS reported to date. Several identified genes are known to be linked with cancer or neurological diseases, many of which have not previously been associated directly to ependymoma. The lack of overlapping common variants or genes between age groups suggests that ependymoma pathogenesis may differ substantially by age at disease onset. Future studies may investigate the functional impact of these susceptibility variants and evaluate their utility as biomarkers for diagnosis and prognosis.

## Supplementary Material

vdag004_Supplementary_Data

## Data Availability

The data sets and computer code used for analyses are available from the corresponding authors upon reasonable request. Genomic and phenotypic study data are available through dbGaP (accession numbers phs001367.v1.p1, phs001327.v1.p1, phs001319.v1.p1), CBTN.org, and St. Jude.Cloud.

## Data Sharing

The Childhood Cancer Survivor Study is a US National Cancer Institute funded resource (U24 CA55727) to promote and facilitate research among long-term survivors of cancer diagnosed during childhood and adolescence. CCSS data are publicly available on dbGaP at https://www.ncbi.nlm.nih.gov/gap/ through its accession number phs001327.v2.p1. and on the St Jude Survivorship Portal within the St Jude Cloud at https://survivorship.stjude.cloud/. In addition, utilization of the CCSS data that leverages the expertise of CCSS Statistical and Survivorship research and resources will be considered on a case-by-case basis. For this utilization, a research Application of Intent followed by an Analysis Concept Proposal must be submitted for evaluation by the CCSS Publications Committee. Users interested in utilizing this resource are encouraged to visit http://ccss.stjude.org. Full analytical data sets associated with CCSS publications since January of 2023 are also available on the St Jude Survivorship Portal at https://viz.stjude.cloud/community/cancer-survivorship-community∼4/publications.
