# Supplementary material for: Discovery of genetic susceptibility variants in pediatric and adult ependymoma
Source: Neurooncol Adv. 2026 Jan 16;8(1):vdag004. doi: 10.1093/noajnl/vdag004 (PMC13000888; doi:10.1093/noajnl/vdag004)

Supplement

S1. Summary of consortia/studies used in analyses

| **Study** | **Location** | **Technology** | **Platform** | ***Age Group** | **Case/Control** |
| --- | --- | --- | --- | --- | --- |
| ACCESS | Texas | Genotyped | Illumina Infinium Global Screening Array | Pediatric/Adult | Case |
| ADD Health^1^ | U.S. | Genotyped | Illumina HumanOmni1-Quad/Illumina HumanOmni2.5 | Pediatric | Control |
| CBTN^2^ | International | WGS | Illumina HiSeq X Ten/Illumina NovaSeq | Pediatric | Case |
| CCSS^3^ | U.S./Canada | Genotyped | Affymetrix Array 6.0 | Pediatric | Case |
| GICC | International | Genotyped | Illumina Infinium OncoArray-500K | Pediatric/Adult | Case/Control |
| NCI-Connect^4^ | International | Genotyped | Illumina Infinium Global Screening Array | Pediatric/Adult | Case |
| St. Jude^5^ | U.S./Canada | WGS | Illumina HiSeq X Ten/Illumina NovaSeq | Pediatric | Case/Control |
| TOPNOC | Texas/Oklahoma | Genotyped | Illumina Infinium Global Screening Array | Pediatric | Case |

Abbreviations: ACCESS – Adolescent and Childhood Cancer Epidemiology and Susceptibility Service of Texas; ADD Health – National Longitudinal Study of Adolescent Health; CBTN – Childhood Brain Tumor Network; CCSS – Childhood Cancer Survivor Study; GICC – Glioma International Case Control Consortium; NCI-Connect – National Cancer Institute’s program for the Comprehensive Oncology Network Evaluating Rare CNS Tumors; St. Jude – St. Jude Cloud; TOPNOC – Texas-Oklahoma Pediatric Neuro-Oncology Consortium; QC – quality control; WGS – Whole Genome Sequenced;

* Age group of cases included in genome-wide association study. Adults were aged 18 years and older, and pediatric individuals were under 18 years old.

1. Resnick MD, Bearman PS, Blum RW, et al. Protecting adolescents from harm. Findings from the National Longitudinal Study on Adolescent Health. Jama. 1997; 278(10):823-832.
2. Lilly JV, Rokita JL, Mason JL, et al. The children's brain tumor network (CBTN) - Accelerating research in pediatric central nervous system tumors through collaboration and open science. Neoplasia. 2023; 35:100846.
3. Leisenring WM, Mertens AC, Armstrong GT, et al. Pediatric Cancer Survivorship Research: Experience of the Childhood Cancer Survivor Study. Journal of Clinical Oncology. 2009; 27(14):2319-2327.
4. Penas-Prado M, Theeler BJ, Cordeiro B, et al. Proceedings of the Comprehensive Oncology Network Evaluating Rare CNS Tumors (NCI-CONNECT) Adult Medulloblastoma Workshop. Neuro-Oncology Advances. 2020; 2(1).
5. McLeod C, Gout AM, Zhou X, et al. St. Jude Cloud: A Pediatric Cancer Genomic Data-Sharing Ecosystem. Cancer Discovery. 2021; 11(5):1082-1099.

S2. Summary of genome-wide association study groups with mixed ancestries

| ***Age Group** | **Genomic Analysis** | **Cohort/Study** | **Case/**  **Control** | **Subjects**  **Pre-QC** | **Subjects**  **Post-QC** | **Sex (male)**  **Post-QC** | **Post-QC Ancestry** | | | | | |
| --- | --- | --- | --- | --- | --- | --- | --- | --- | --- | --- | --- | --- |
|  |  |  |  |  |  |  | **AMR** | **AFR** | **EAS** | **EUR** | **SAS** |  |
| **Pediatric** | Genotyping | ACCESS | Case | 87 | 87 | 62 | 12% | 5% | 3% | 79% | 1% |  |
|  |  | CCSS |  | 58 | 58 | 27 |  |  |  |  |  |  |
|  |  | GICC |  | 8 | 8 | 4 |  |  |  |  |  |  |
|  |  | NCI-Connect |  | 10 | 9 | 5 |  |  |  |  |  |  |
|  |  | TOPNOC |  | 19 | 18 | 13 |  |  |  |  |  |  |
|  |  | ADD Health | Control | 2349 | 2340 | 1116 | 13% | 24% | 5% | 57% | 1% |  |
|  | Whole Genome Sequencing | CBTN | Case | 65 | 65 | 39 | 9% | 11% | 4% | 87% | 1% |  |
|  |  | St. Jude |  | 100 | 92 | 42 |  |  |  |  |  |  |
|  |  | St. Jude | Control | 343 | 329 | 156 | 4% | 24% | 1% | 75% | 1% |  |
| **Adult** | Genotyping | ACCESS | Case | 6 | 6 | 3 | 0% | 0% | 0% | 100% | 0% |  |
|  |  | GICC |  | 77 | 73 | 40 |  |  |  |  |  |  |
|  |  | NCI-Connect |  | 157 | 140 | 45 |  |  |  |  |  |  |
|  |  | GICC | Control | 3249 | 3225 | 1842 | 0% | 0% | 0% | 100% | 0% |  |

Abbreviations: ACCESS – Adolescent and Childhood Cancer Epidemiology and Susceptibility Service of Texas; ADD Health – National Longitudinal Study of Adolescent Health; AFR – African; AMR – Admixed-American; CBTN – Childhood Brain Tumor Network; CCSS – Childhood Cancer Survivor Study; EAS – East Asian; EUR – European; GICC – Glioma International Case Control Consortium; NCI-Connect – National Cancer Institute’s program for the Comprehensive Oncology Network Evaluating Rare CNS Tumors; SAS – South Asian; St. Jude – St. Jude Cloud; TOPNOC – Texas-Oklahoma Pediatric Neuro-Oncology Consortium; QC – quality control

* Age group of cases included in genome-wide association study. Adults were aged 18 years and older, and pediatric individuals were under 18 years old.

S3. Genome-wide association study of whole-genome sequenced pediatric ependymoma of mixed ancestry. Each genome-wide association study displays the corresponding quantile-quantile plot (left) and Manhattan plot (right). The red dotted horizontal line indicates the genome-wide significance threshold (P=5×10^-8^), and the green dotted line marks the suggestive significance threshold (P=1×10^-5^).


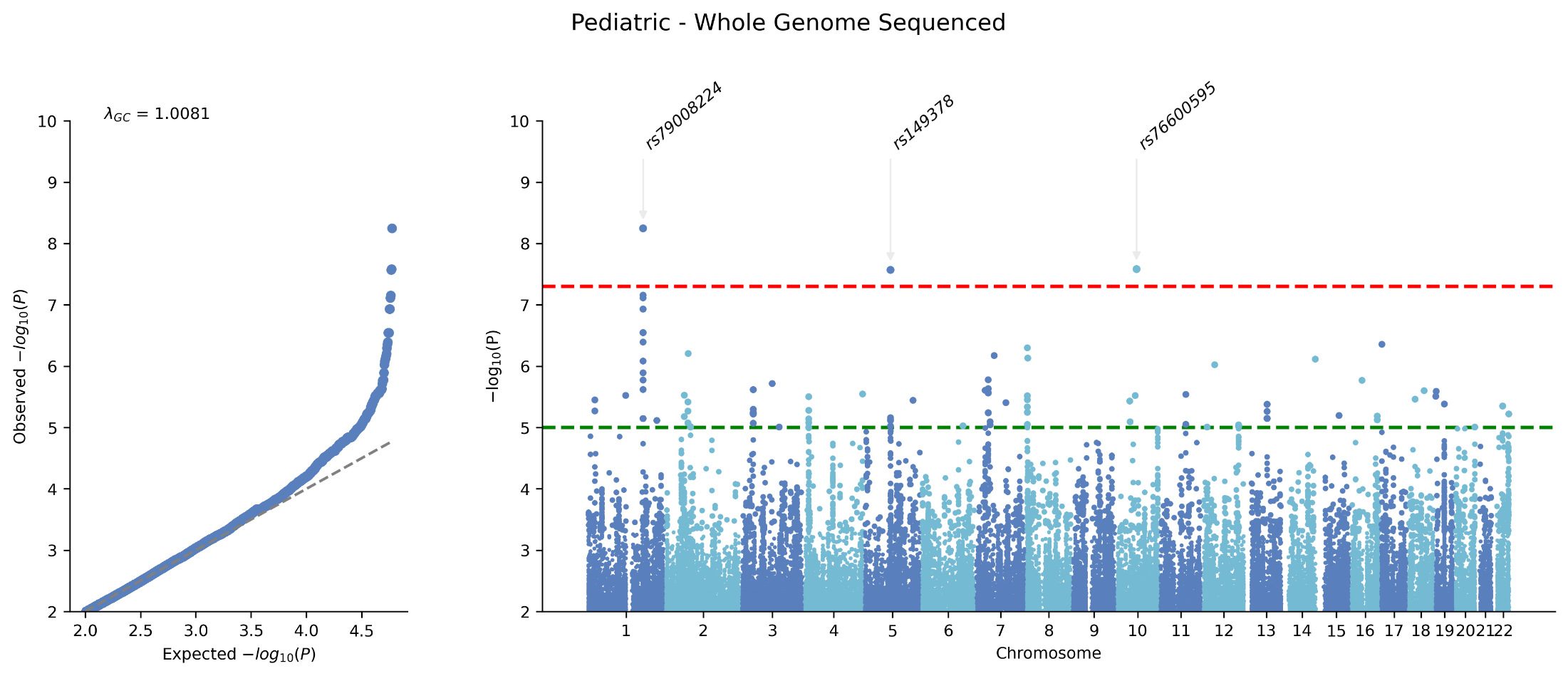


S4. Genome-wide association study of genotyped pediatric ependymoma of mixed ancestry. Each genome-wide association study displays the corresponding quantile-quantile plot (left) and Manhattan plot (right). The red dotted horizontal line indicates the genome-wide significance threshold (P=5×10^-8^), and the green dotted line marks the suggestive significance threshold (P=1×10^-5^).


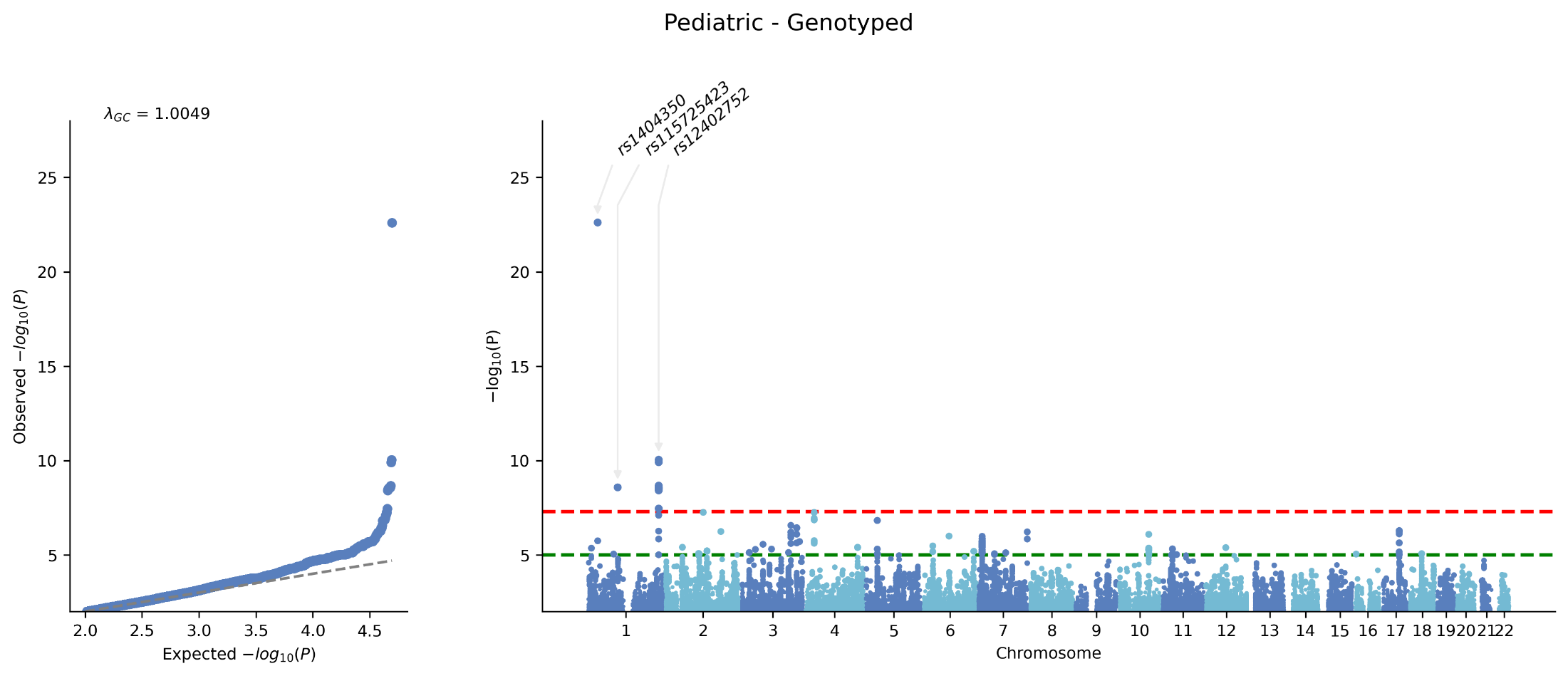

Supplement: vdag004_Supplementary_Data [file vdag004_supplementary_data.zip › Supplement.docx]
